# Sedation as an Immunomodulator of Inflammatory Responses in the Lung–Brain Axis of ARDS

**DOI:** 10.3390/ijms27114700

**Published:** 2026-05-23

**Authors:** Cassian-Gabriel Gălbenușe, Andreea Doriana Stănculescu, Nicoleta Alice Drăgoescu

**Affiliations:** 1 Faculty of Medicine, University of Medicine and Pharmacy of Craiova, 200349 Craiova, Romania; cassiangalbenuse@yahoo.ro; 2 Department of Anesthesiology and Intensive Care, Emergency County Hospital of Craiova, 200642 Craiova, Romania

**Keywords:** neuroinflammation, ARDS, delirium, immunomodulation, sedative agents

## Abstract

Acute respiratory distress syndrome (ARDS) is characterized by systemic inflammation, immune dysregulation, oxidative stress, and frequent extrapulmonary organ involvement. Neurological complications of ARDS, such as neuroinflammation, cognitive impairment and delirium, are common and worsen outcomes. Early evidence highlights bidirectional communication between the lungs and brain, the lung–brain axis, through which inflammation may amplify both pulmonary and cerebral injury. This narrative review synthesizes recent experimental and clinical data on the immunomodulatory and neuroprotective effects of commonly used sedative agents in ARDS, focusing on their influence on inflammatory mediators (IL-1β, IL-6, IL-8, IL-10, TNF-α) and neuronal injury biomarkers (S100B, neuron-specific enolase). Sedative agents seem to exert effects beyond sedation by modulating systemic and neuroinflammatory responses. Evidence suggests they can influence cytokine profiles and reduce biomarkers associated with neuronal injury, potentially mitigating neuroinflammation and delirium in ARDS patients. Sedatives may modulate lung–brain crosstalk in ARDS through immunoinflammatory pathways, integrating sedative and neuroprotective effects. Mechanistic clarification may enable targeted sedation strategies to improve pulmonary and neurological outcomes.

## 1. Introduction

Acute respiratory distress syndrome (ARDS) is a complex and heterogeneous clinical entity characterized by acute inflammatory lung injury, non-cardiogenic pulmonary edema, and severe hypoxemic respiratory failure [1,2]. Beyond the pulmonary compartment, ARDS is increasingly recognized as a systemic inflammatory syndrome with extrapulmonary consequences, including potential involvement of the central nervous system.

Accumulating evidence suggests that systemic inflammation, hypoxemia, endothelial dysfunction, and ventilator-associated stress may contribute to blood–brain barrier disruption and neuroinflammatory processes, providing a biological basis for lung–brain crosstalk in critically ill patients [3]. Clinically, these mechanisms may manifest as acute brain dysfunction, including delirium, and contribute to long-term cognitive impairment [4].

The management of ARDS relies on supportive strategies such as lung-protective mechanical ventilation, analgesia, and sedation [5]. However, sedation in this context is not a neutral intervention. Depth of sedation, duration, and choice of agent may variably influence neurological outcomes, yet their role in modulating inflammatory and neuroinflammatory pathways remains insufficiently defined [6,7,8].

Although increasing experimental and translational evidence suggests that sedative agents may exert immunomodulatory and neuroprotective effects [9,10,11], their integration into a coherent framework linking lung injury, systemic inflammation, and neuroinflammation in ARDS remains limited.

In an international cohort analysis of 1450 neurocritically ill patients, initial sedation strategies on day one were predominantly comprised of propofol (41.2%), followed by midazolam (26.1%), a combination of propofol and midazolam (19.9%), no sedation (11.8%), dexmedetomidine (0.7%), and sodium thiopental (0.3%) [12]. While volatile anesthetics [13,14,15,16,17,18,19,20,21,22,23] and opioids [24,25,26,27,28,29,30,31] have also demonstrated intrinsic anti-inflammatory and immunomodulatory properties, their clinical application in the ARDS context is often primary to anesthesia or analgesia rather than primary, long-term sedation. Consequently, to ensure a high-resolution analysis of the most clinically prevalent sedation regimens, this review is specifically delimited to the previously mentioned intravenous agents, focusing on their distinct roles in modulating the lung–brain axis [32,33,34].

In this narrative review, we examine whether the commonly used sedative agents may influence the lung–brain axis in ARDS through immunomodulatory, anti-inflammatory, and neuroprotective mechanisms, while critically distinguishing mechanistic evidence from clinically actionable evidence. Our hypothesis may serve as a framework for future research aimed at determining whether conventional sedatives used in intensive and neurocritical care settings possess pleiotropic effects, specifically in modulating the neuroinflammatory cascade associated with ARDS.

## 2. Methods and Search Strategy

A structured literature search was performed in major electronic databases, including PubMed/MEDLINE, Scopus, and Web of Science, covering publications form January 2016 to January 2026. To find the most relevant scientific papers, a combination of keywords was used, including “acute respiratory distress syndrome” OR “ARDS”, “sedation”, “sedative agents”, “propofol”, “dexmedetomidine”, “benzodiazepines”, “ketamine”, “neuroinflammation”, “lung–brain axis”, and “cytokines”, using Boolean operators (AND, OR).

The review focused primarily on peer-reviewed publications from high-quality journals, with an emphasis on the recent literature. Studies were selected based on their relevance to the relationship between ARDS, neuroinflammation, and the immunomodulatory effects of sedative agents. Priority was given to experimental (in vitro and animal), translational, and clinical studies that provided mechanistic or clinically applicable insights. Whenever possible, ARDS-specific evidence was prioritized. However, in the absence of direct data, relevant studies from other ICU-related conditions characterized by systemic inflammation (e.g., sepsis, perioperative critical illness, neurocritical care) were included to support mechanistic understanding. A series of studies prior to 2016 were incorporated to provide a longitudinal perspective on the evolution of evidence regarding sedative agents, including their pharmacological properties and broader clinical utility; the older studies were included selectively when they contributed foundational or otherwise unavailable information relevant to the topic. Additional relevant publications were identified through manual screening of the reference lists of included articles.

Exclusion criteria comprised studies not addressing the anti-inflammatory, immunomodulatory, or neuroinflammatory effects of sedative agents, as well as those lacking relevance to ARDS or critical illness. Non-English publications were excluded. Studies focused specifically on COVID-19–related ARDS were selectively excluded to minimize pathophysiological heterogeneity unless they provided fundamental mechanistic insights applicable to non-viral ARDS. Articles lacking clear conclusions or interpretative value regarding sedative-related effects were also excluded. Studies evaluating the anti-inflammatory or immunomodulatory effects of volatile anesthetics and opioids were excluded. Given the narrative nature of this review, no formal systematic review protocol was applied. However, efforts were made to ensure a comprehensive and balanced representation of the available evidence, focusing on key mechanisms linking pulmonary and neuroinflammation and the potential impact of sedative strategies.

## 3. ARDS as a Systemic Inflammatory Syndrome

ARDS can be conceptualized as a systemic immuno-inflammatory syndrome initiated by acute injury to the alveolar–capillary barrier. Disruption of both the alveolar epithelium and capillary endothelium leads to increased permeability, non-cardiogenic pulmonary edema, and impaired gas exchange. This local injury triggers a robust inflammatory response characterized by the recruitment and activation of neutrophils, the release of pro-inflammatory cytokines, and amplification of oxidative and endothelial damage. In parallel, endothelial dysfunction promotes leukocyte adhesion, platelet activation, and microvascular thrombosis, contributing to ventilation–perfusion mismatch and further tissue injury [35,36,37,38,39,40].

Importantly, these processes are not confined to the lung. The systemic spillover of inflammatory mediators, together with endothelial injury and ventilator-associated stress, may propagate a broader inflammatory response affecting distant organs. In this context, the brain represents a particularly vulnerable target. Circulating cytokines, hypoxemia, and microvascular dysfunction may contribute to blood–brain barrier disruption and activation of neuroinflammatory pathways, thereby establishing the biological substrate for lung–brain crosstalk in ARDS [3,41,42].

## 4. Pathophysiology of ARDS-Associated Neuroinflammation

Brain dysfunction represents a frequent and clinically relevant extrapulmonary manifestation of ARDS, encompassing delirium, cognitive impairment, and long-term neurocognitive decline [43,44]. A recent review mentioned high rates of secondary acute brain injury (e.g., hemorrhagic stroke, hypoxic–ischemic injury) in the ARDS population, with poor neurological outcomes in 82–86% of some cohorts [45]. Cognitive impairment is extremely common at discharge (≈80–100%), and persists in ~30–40% at 6–12 months, affecting memory, attention, and processing speed [43,45]. These complications are likely multifactorial, arising from the interaction between systemic inflammation, hypoxemia, hemodynamic instability, and intensive care–related exposures, including mechanical ventilation and sedation.

### 4.1. The Current Literature Knowledge

The relationship between the lungs and brain seems to be bidirectional [46,47]: lung injury and ventilator induced lung injury (VILI) may lead to brain impairment and cognitive alterations [45,48,49], while primary brain injury can lead to pulmonary damage [50]. The current literature extensively discusses the brain–lung axis, primarily through frameworks such as the “double-hit” [51] or “triple-hit” hypotheses [52]. The most established model, the “double-hit” theory, suggests that a primary acute brain injury (ABI) triggers a systemic inflammatory surge. These circulating cytokines, following inflammatory response, reach extracranial organs via the bloodstream, with the lungs being particularly vulnerable. The “second hit” typically involves the direct effects of ABI—such as systemic inflammation and oxidative stress—which exacerbate the mechanical stress of ventilation or the presence of infection. Some authors further propose a “triple-hit” model, incorporating intestinal dysfunction and microbiome alterations in ABI patients, thereby engaging the brain–gut–lung axis [53]. Crucially, these mechanisms are inherently bidirectional. Just as neuroinflammation can drive pulmonary injury, it is mechanistically and biologically plausible that a profound inflammatory syndrome like ARDS can act as the primary insult, leading to secondary cerebral inflammation [3,41,43]. Recognizing this inverse lung-to-brain pathway is essential for understanding how pulmonary pathology translates into neurological dysfunction.

### 4.2. Mechanistic Plausibility

Several interconnected mechanisms have been proposed to explain ARDS-associated neuroinflammation. Elevated levels of IL-6, IL-1β, and TNF-α may correlate directly with blood–brain barrier (BBB) disruption and subsequent microglial activation; in particular, IL-6 is increasingly recognized as a potential key mediator in signaling along the lung–brain axis [54], suggesting that these systemic cytokine fluctuations might serve as crucial conduits for downstream neuroinflammatory processes. In parallel, hypoxemia and microvascular dysfunction may further exacerbate neuronal vulnerability and impair neurotransmission [43,55]. BBB dysfunction appears to play a central role in this process. Increased permeability facilitates the passage of inflammatory mediators and immune cells into the brain, promoting microglial activation and amplification of local inflammatory cascades. These processes have been associated with neuronal injury, apoptosis, and the development of acute brain dysfunction in critically ill patients [43,44,50,56,57].

A growing body of recent research bridges the gap between experimental observations and clinical applications, shedding light on the intricate molecular processes involved. Specifically, alveolar epithelial cells have been shown to secrete extracellular vesicles (EVs) enriched with miR-106a-5p, which cross the blood–brain barrier (BBB) and activate the MAPK signaling pathway in the brain, thereby contributing to cognitive dysfunction [58]. Furthermore, evidence indicates that protein tyrosine phosphatase receptor type O (PTPRO) in brain endothelial cells regulates HIF-1α-dependent glycolysis during sepsis, ultimately leading to BBB disruption and neutrophil infiltration [59]. In addition, pulmonary cytokines directly stimulate vagal afferent fibers, transmitting inflammatory signals to the nucleus tractus solitarius in the brainstem, which are subsequently projected to cortical regions involved in cognition and emotion [60,61]. Interestingly, a pivotal mechanism driving barrier disruption involves the degradation of tight junction proteins (such as Claudin-5, Occludin, and ZO-1) by matrix metalloproteinases (MMP-9) that are systemically activated by lung inflammation [61,62,63].

### 4.3. Translational Biomarkers

A number of biomarkers have been investigated to support this mechanistic framework. Proteins such as S100B, neuron-specific enolase (NSE), and markers of endothelial activation have been associated with both pulmonary and neurological injury [64]. In particular, the S100B/RAGE signaling axis has been proposed as a potential link between systemic inflammation and neuroinflammatory amplification, including interactions with neutrophil extracellular traps (NETs) in experimental models. Furthermore, animal models of ARDS have been shown to demonstrate a significant increase in S-100β protein expression, which in this context appears to be associated with the atrophy of pyramidal neurons in the hippocampal CA1 region [54]. Similarly, elevated plasma levels of NfL are suggested as potentially robust indicators of axonal injury within the framework of pollutant exposure and chronic pulmonary pathologies [61]. NfL has, in turn, been proposed for the evaluation of encephalopathy secondary to sepsis, a frequent underlying cause of ARDS [65]. Additionally, increased plasma levels of GFAP may reflect heightened astrocytic reactivity, rendering it a tentative biomarker for detecting “silent” neuroinflammation prior to the onset of irreversible neurodegeneration [61]. However, these findings are derived predominantly from preclinical and translational studies [41,66,67,68,69,70,71].

Clinical studies report that, in ARDS and traumatic brain injury (TBI), several biomarkers such as DAMPs (eNAMPT, S100A8), cytokines (IL-6, IL-1β, TNF-α), and neurotrauma markers (GFAP, tau, NFL) are elevated, with further increases in mechanically ventilated patients, linking lung injury and neurotrauma biology. Furthermore, by targeting TNF-α or eNAMPT, lung–brain inflammatory signaling may be downregulated in ARDS models and early clinical trials [41]. Utilized in post-COVID-19 monitoring studies, with COVID-19 frequently considered a phenotype of ARDS, the DTI-ALPS index appears to reflect dynamic changes in the glymphatic system and has been shown to correlate negatively with cognitive scores [60,72]. Furthermore, acute hypoxia in ALI is observed to stabilize the HIF-1α factor within the brain; this process is accompanied by increases in lactate and reactive oxygen species (ROS), which may serve as metabolic biomarkers indicative of a neuronal energetic crisis [54,59]. Additionally, the recent literature suggests that the downregulated expression of tight junction proteins, such as ZO-1, Claudin-5, and Occludin, within brain endothelial cells represents a key molecular hallmark of potential BBB compromise driven by pulmonary cytokines [54,73]. Ultimately, the early identification of these biomarkers, particularly NfL and GFAP, during routine evaluations of patients with severe respiratory distress might facilitate the detection of subclinical neuroinflammation prior to the onset of permanent cognitive decline.

### 4.4. Implications of Mechanical Ventilation

It is well-established that mechanical ventilation, while being a cornerstone in critical care, can cause direct pulmonary damage or exacerbate underlying lung injury especially in conditions such as ARDS. The literature supports the premise that the ventilator induces significant strain and stress on lung tissue, leading to ventilator-induced lung injury (VILI) [47,74]. This mechanical insult initially manifests as a localized inflammatory response, which subsequently progresses to a systemic spillover of cytokines into the circulation, further reaching the BBB level, which the inflammatory mediators will destroy, and causes brain damage. Elevated levels of circulating inflammatory and neuronal injury biomarkers, such as neurofilament light chain (NFL), have been seen in critically ill patients requiring prolonged ventilatory support [41,75]. Furthermore, VILI has been implicated in triggering neuronal hyperexcitability, ultimately leading to brain cell apoptosis. Thus, mechanical ventilation alone seems to induce hippocampal apoptotic pathways and early cerebral inflammation, even without established lung injury [75]. This neuronal destruction is further exacerbated by the repetitive stretching of alveolar cells and the resulting inflammation, which triggers a vagal response back to the brain [50,76,77,78]. Collectively, these mechanisms form the clinical entity known as ventilator-associated brain injury (VABI) [48,79,80], a concept strongly supported by evidence from preclinical studies [81,82,83].

Lung-protective low tidal volume ventilation, recommended in ARDS [5,84], can cause hypercapnia, cerebral vasodilation, increased cerebral blood flow and intracranial pressure (ICP), potentially worsening ischemic injury [45]. Conversely, studies suggest that a low tidal volume strategy improves brain oxygenation and reduces cytokine release when compared with high tidal volume strategy [44].

**Figure 1:**
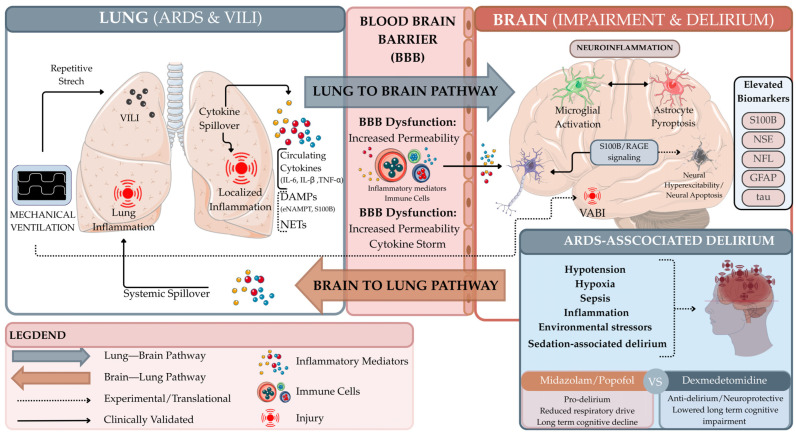
Conceptual model of ARDS-induced neuroinflammation pathway. ABI, acute brain injury; ARDS, acute respiratory distress syndrome; BBB, blood–brain barrier; DAMPs, damage-associated molecular patterns (e.g., eNAMPT, S100A8); GFAP, glial fibrillary acidic protein; IL, interleukin; NETs, neutrophil extracellular traps; NFL, neurofilament light chain; NSE, neuron-specific enolase; RAGE, receptor for advanced glycation end-products; S100B, S100 calcium-binding protein B; TNF- α, tumor necrosis factor-alpha; VABI, ventilator-associated brain injury; VILI, ventilator-induced lung injury.

Overall, the connection between the lung and brain is complex and includes inflammation, immune suppression, and neurodegeneration [3,49,74,85]. Importantly, direct clinical evidence linking specific lung-derived inflammatory pathways to neuroinflammation in ARDS remains limited. Much of the current understanding is based on experimental models, in vitro data, and extrapolation from related critical care conditions, and should therefore be interpreted as hypothesis-generating rather than definitive (Figure 1).

## 5. ARDS-Associated Delirium

ARDS is frequently complicated by acute brain dysfunction, with delirium representing the most common neurological manifestation in critically ill patients. This association reflects not only the severity of the underlying disease but also the cumulative impact of intensive care–related exposures, including invasive mechanical ventilation, sedation, and neuromuscular blockade [86,87]. Mortality in ARDS remains high, and a substantial proportion of survivors develop long-term neurocognitive impairment, with delirium acting as a key intermediate clinical phenotype [86].

Delirium is defined as an acute and fluctuating disturbance of attention and awareness and is associated with increased healthcare costs, prolonged hospitalization, and long-term cognitive decline [88,89,90,91]. Importantly, delirium in ARDS should be viewed as a multifactorial syndrome rather than a single-pathway phenomenon.

From a biological perspective, neuroinflammation represents a major contributing mechanism. Elevated circulating inflammatory markers, including IL-6, IL-8, TNF-α, and C-reactive protein (CRP), have been consistently associated with the development and severity of delirium in critically ill patients [92,93,94]. These findings support the hypothesis that systemic inflammation may influence brain function through cytokine-mediated pathways, contributing to altered neurotransmission and neuronal dysfunction. However, inflammation alone does not fully explain the occurrence of delirium. Clinical and translational data suggest the existence of distinct delirium phenotypes, including metabolic, hypoxic, septic, and sedative-associated forms [95]. Among these, sedative-related delirium is particularly relevant, as it represents a potentially modifiable contributor to acute brain dysfunction.

In this context, sedation should not be viewed solely as a supportive intervention but rather as a factor that may interact with underlying inflammatory and physiological processes. The depth, duration, and pharmacological profile of sedative agents may influence the risk, severity, and trajectory of delirium, especially in patients with ARDS and systemic inflammation [87]. Therefore, sedatives can both promote and mitigate delirium, and delirium itself often reflects the underlying severity of ARDS and critical illness. Evidence links drug class, depth and duration of sedation, and ARDS stage with delirium risk and longer-term cognitive outcomes.

### 5.1. Pro-Delirium and Anti-Delirium Mechanisms

Both midazolam and propofol reduce respiratory drive and can induce deep sedation, but benzodiazepines are associated with a higher risk of developing delirium. Propofol is preferred over midazolam because it is less likely to cause prolonged sedation or delirium and is easier to titrate [24]. A high cumulative benzodiazepine dose was linked to 41% higher 90-day mortality in a large cohort [96], and prolonged sedative exposure in ARDS is a risk factor for delirium and long-term cognitive decline [44].

Dexmedetomidine may be associated with a lower prevalence of delirium than midazolam or propofol in some RCTs and delayed time to delirium in cohort data [97,98]. Furthermore, animal and immunology papers suggests anti-inflammatory and neuroprotective actions [99,100]. Moreover, dexmedetomidine-based sedation in polytrauma reduced delirium rates and lowered brain injury biomarkers (S100B, NSE, BDNF) versus midazolam/propofol regimens [101].

### 5.2. Sedation-Associated Versus Disease-Associated Delirium

Delirium often arises from acute pathophysiology, such as hypotension, hypoxia, sepsis, inflammation, and environmental stressors [102], and is strongly associated with long-term cognitive impairment after ARDS [44]. However, sedation seems to be an independent contribuitor [43]. Deep or prolonged sedation is linked to more delirium or fewer delirium-free days and worse function, even after adjusting for illness severity, in multiple cohorts and intervention studies [8,96,97]. The ABCDEF/ABCDEF-R bundles explicitly aim to reduce sedative exposure, allow early mobilization, and systematically assess delirium to prevent both sedation- and disease-driven delirium [24,97]. In acute lung injury, David N. Hager et al. created and used a new protocol that minimized continuous infusions and targeted RASS 0 which markedly reduced benzodiazepine exposure and increased days awake and free of delirium [8].

### 5.3. Deep Compared to Light Sedation in Different ARDS Stages

Evidence notes that deep sedation and sometimes paralysis are often required in severe ARDS, especially early, to allow lung-protective ventilation. However, minimizing depth and duration remains a goal [24]. Conversion to light sedation should be made as soon as feasible, and deep sedation should be used with caution following clear indications in mechanically ventilated patients with ARDS [97]. Therefore, deep sedation, often with GABAergic drugs, may be necessary for lung protection but carries independent risks of mortality, delayed extubation, and potentially delirium and long-term cognitive harm if prolonged [44,103]. Evidence across ARDS/ALI and general ICU populations supports early transition to light sedation, reduced benzodiazepines, daily sedative interruption, and mobilization to increase delirium-free days and possibly survival [8,97,104].

Another important aspect is the association between sedation depth and illness severity. In a meta-analysis of 18 studies (8001 ventilated adults), deep vs light sedation was not associated with delirium in RCTs (OR ≈ 1.0), even after accounting for benzodiazepine dose and disease severity [102], but still suggested more delirium with lighter sedation, likely reflecting confounding by indication.

Guidelines and ARDS reviews state that benzodiazepines carry a higher delirium risk, whereas propofol as a first-line treatment, along with dexmedetomidine added to reduce emergent delirium and cumulative dose, is favored when feasible [24]. Moreover, RCTs outside ARDS show dexmedetomidine compared to lorazepam/midazolam increases delirium- and coma-free days at similar targeted RASS, suggesting a drug-class effect independent of nominal depth [9,97]. Within RCTs, adjusting for different BDZ doses did not change the association between depth and delirium [102]. However, cumulative exposure can be reduced by using guided sedation methods. BIS-guided deep sedation reduced propofol and midazolam doses and, in patients sedated >24 h, increased delirium- and coma-free days compared to clinical assessment at a similar targeted depth, implying that avoiding overshoot exposure at the same depth can favorably affect brain outcomes [105,106].

Overall, disease-associated delirium is driven by inflammatory and vascular injury, while sedation-associated delirium is layered on top, strongly influenced by drug class and depth/exposure.

## 6. Pharmacologic Modulation of the Lung–Brain Axis

The interplay between sedative agents, pulmonary physiology, and neuroprotection is increasingly recognized as a critical factor in the management of critically ill patients, particularly those requiring mechanical ventilation or suffering from acute brain injury. Sedatives such as dexmedetomidine and propofol have demonstrated not only direct neuroprotective effects but also the ability to modulate pulmonary inflammation, improve lung compliance, and reduce the cytokine overflow mechanism that may protect the brain via the lung–brain axis [50,56,80,107,108,109,110,111,112]. Evidence from both animal models and clinical studies suggests that optimizing sedation strategies can attenuate systemic inflammation, preserve the blood–brain barrier integrity, and decrease neuronal injury markers, thereby linking improved pulmonary outcomes to downstream neuroprotection [48,109,110,111,112]. Indeed, the relationship is complex. While some sedatives confer benefits through anti-inflammatory pathways, others may exacerbate neuroinflammation or cognitive impairment if not carefully managed [44,113,114] (Table 1).

Sedatives such as dexmedetomidine and propofol have been shown to reduce pulmonary inflammation (e.g., TNF-α, IL-6), improve compliance, decrease histologic lung injury scores, and limit cytokine spillover in models of acute ischemic stroke or sepsis [109,110,111]. Dexmedetomidine demonstrated bronchodilator properties and reduced airway constriction compared to propofol or ketamine [110,111]. Propofol also exhibited anti-inflammatory effects but was less potent than dexmedetomidine in some models [161].

Mechanical ventilation or acute lung injury can trigger the systemic release of inflammatory cytokines (IL-6, TNF-α), which cross the blood–brain barrier and induce neuronal apoptosis or cognitive dysfunction, translated into VABI [50,56,80]. Sedation strategies that minimize lung injury or inflammation can reduce this cytokine-mediated brain insult [109,110,112]. Dexmedetomidine consistently reduced markers of neuronal damage (e.g., S100β), preserved BBB integrity, decreased microglial activation, inhibited apoptosis pathways (caspase-3), and improved cognitive outcomes in both animal models and clinical settings [108,109,110,111,112,240,241]. Propofol also showed neuroprotective effects by inhibiting microglial activation through multiple signaling pathways, for example PI3K/Akt/mTOR [107,172].

### 6.1. Dexmedetomidine

Dexmedetomidine (DEX) is a highly selective α2-adrenergic receptor agonist widely used as an adjunctive sedative in critically ill patients, particularly in mechanically ventilated ICU populations, as well as in neurocritical care settings where its potential neuroprotective properties are of interest [115,242]. Beyond its sedative effects, dexmedetomidine seems to exert significant immunomodulatory activity.

#### 6.1.1. Preclinical Mechanistic Evidence: Animal/In Vitro Models

Dexmedetomidine consistently reduced pro-inflammatory cytokines (TNF-α, IL-1β, IL-6) in animal models of ARDS/ALI/sepsis [127,141,144], with additional suppression of MIP-2 [121], iNOS/NO [118], NF-κB activation [134,139,147], NLRP3 inflammasome activity [119,140], HMGB1 expression [138], TLR4 signaling, PI3K/Akt/mTOR pathway activation [116], ERK1/2 phosphorylation [139], oxidative stress markers (MDA↓/SOD↑) [130,131], microglial activation [140], astrocyte pyroptosis [146], and blood–brain barrier disruption [147]. These effects were often reversed by α2-antagonists or pathway-specific inhibitors. Overall, mechanistically coherent links are established between α2-adrenoceptor activation and downstream suppression of the TLR4/NF-kB/NLRP3/Akt/mTOR pathways, resulting in decreased systemic inflammation, resulting in lung injury attenuation, neuroinflammation reduction through microglia/astrocyte modulation, and improved blood–brain barrier integrity/neuroprotection/cognitive outcomes across models ranging from LPS-induced ALI/ARDS to sepsis-associated encephalopathy/postoperative cognitive dysfunction/TBI/stroke models.

#### 6.1.2. Translational Biomarker Evidence: Human/Clinical Biological Signals

In perioperative patients and those with sepsis or ARDS requiring mechanical ventilation, dexmedetomidine reduced plasma levels of IL-6, TNF-α, IL-8, CRP, S100B protein, NSE (neuron-specific enolase), cortisol and glucose surges post-injury/surgery [120,123,137,145]. Also, studies reported increased anti-inflammatory cytokines such as IL-10 [123]. Nevertheless, direct evidence for all pathways and biomarkers specifically within human ARDS populations is limited, as most mechanistic details comes from animal or in vitro studies.

#### 6.1.3. Randomized Clinical Evidence

Meta-analyses of RCTs confirm that perioperative or ICU administration of dexmedetomidine significantly decreases circulating levels of IL-6, TNF-α, and IL-8 immediately after surgery or on postoperative day one and increases IL-10. Also, DEX has been shown to reduce S100B/NSE levels, improve MMSE scores, and lower the incidence of postoperative cognitive dysfunction, but it does not consistently affect CRP or long-term mortality across all settings [120,122,123,136,145]. However, clinical trials show consistent biomarker changes but variable translation into clinical outcomes such as mortality or long-term cognition.

### 6.2. Propofol

Propofol is a phenol-derived anesthetic agent widely used in neurocritical care due to its ability to reduce cerebral metabolic rate and intracranial pressure, making it a preferred sedative in patients with various neurological impairments [242]. Beyond its hemodynamic and neurophysiological effects, propofol has been shown to modulate immune function through multiple mechanisms.

#### 6.2.1. Preclinical Mechanistic Evidence

Propofol consistently inhibits microglial activation via downregulation of the PI3K/Akt pathway—reducing production of NO, ROS, TNF-α—and suppresses NF-κB-mediated transcription of inflammatory mediators [107,157,172]. It also upregulates miR-106b to inhibit PI3K/Akt signaling [157], modulates the miR-221/222–IRF2 axis [160], suppresses TGM2/NF-κB signaling [167], regulates the miR-155/SOCS1 pathway [163], maintains the Th17/Treg balance via miR-145-3p/NFATc2/NF-κB [173], inhibits MMP-9 expression through the Ca^2+^/CAMKII/ERK/NF-κB pathway [171], activates Nrf2/HO-1 to combat ferroptosis [164], inhibits NLRP3 inflammasome activation [159], reduces oxidative stress by scavenging ROS/myeloperoxidase activity [174], and attenuates metabolic reprogramming in microglia [107]. These effects are demonstrated primarily in animal models or cell cultures simulating ARDS or related inflammatory states. However, the modulatory activity of propofol seems to be stronger at the level of microglial polarization, favoring the M2 phenotype, metabolic reprogramming inhibition via ROS/PI3K/Akt/mTOR/HIF-1α, ferroptosis prevention though the Nrf2/HO-1 pathway, and direct antioxidant action [107,164,174].

#### 6.2.2. Translational Biomarker Evidence

Animal studies suggest that propofol reduces pro-inflammatory cytokines (TNF-α, IL-1β, IL-6) in alveolar macrophages or bronchoalveolar lavage fluid during lung injury models [99,154]. In addition, it decreases MMP-9 expression in cerebral endothelial cells exposed to TNF-α [171]. Propofol increases the anti-inflammatory cytokine IL-10 locally while suppressing IL-6/IL-8 during surgical stress [153]. However, prior studies report increased systemic levels of certain cytokines after prolonged infusion in critically ill patients [176].

#### 6.2.3. Observational and Randomized Clinical Evidence

Clinical studies provide mixed results. Propofol may reduce neuroinflammation by suppressing microglial activation but has been associated with increased S100B levels (a marker for BBB disruption) compared to dexmedetomidine in some ICU cohorts [148]. In perioperative settings, propofol is linked to a reduced incidence of cognitive decline compared to inhaled agents but does not consistently lower systemic inflammatory markers relative to sevoflurane or midazolam [156,158].

### 6.3. Benzodiazepines

Although benzodiazepines are increasingly avoided in the sedation of critically ill patients due to their association with delirium, cognitive impairment, withdrawal syndromes, prolonged mechanical ventilation, and increased ICU length of stay [242], several experimental studies have suggested that this class of drugs may exert immunomodulatory and anti-inflammatory effects [181]. However, the overall evidence remains inconsistent and largely derived from preclinical models.

Notably, benzodiazepines can suppress pro-inflammatory cytokine release in both lung and brain models of injury or infection [178,193,194,195], reduce neuroinflammatory markers [179,182,189,190,191], inhibit oxidative stress [189,197], and modulate cognitive impairment in sepsis or ARDS models [50,189]. However, some evidence suggests the potential for immunosuppression or adverse neurocognitive outcomes with prolonged use [183,184,185]. The mechanistic coherence of these effects is supported by studies linking TSPO activation to anti-inflammatory responses in microglia and macrophages [186,188,193], suppression of NLRP3 inflammasome-mediated pyroptosis [191], inhibition of NF-κB/MAPK signaling [193,195], modulation of STAT pathways [194], and reduction of HMGB1 expression in lung injury [192]. In addition, midazolam has been shown to modulate innate immune cell function, including inhibition of macrophage oxidative burst, as well as suppression of neutrophil and mast cell activity [34]. These effects are associated with reduced secretion of pro-inflammatory cytokines such as interleukin-6 (IL-6) and tumor necrosis factor-alpha (TNF-α). Given that IL-6 is closely linked to neuroinflammation, cognitive dysfunction, and delirium, its downregulation may have potential clinical relevance [34,193]. Moreover, TNF-α plays a key role in blood–brain barrier (BBB) permeability, synaptic transmission, and plasticity, and its modulation may influence neuroinflammatory cascades.

#### 6.3.1. Preclinical Mechanistic Evidence

Midazolam suppresses LPS-induced upregulation of CD80 and release of IL-6/TNF-α/nitric oxide in human macrophages via TSPO activation. This effect is confirmed by TSPO ligands and knockdown studies [193]. In animal models of neuroinflammation, diazepam reduces CNS inflammatory cell infiltration and TSPO expression [179]. In rat microglia cultures exposed to LPS or injury mimics, benzodiazepines decrease NO/TNF-α release via PBR/TSPO engagement [186]. Midazolam inhibits LPS-induced p38 MAPK phosphorylation and NF-κB activation in macrophages—reducing iNOS/COX2 expression—and suppresses superoxide production [195]. In glial cells stimulated with IL-1β (a key driver of neuroinflammation), midazolam inhibits STAT3 phosphorylation and IL-6 release without affecting p38 MAPK/SAPK/JNK/IκB phosphorylation—suggesting selective pathway targeting [194].

Remimazolam reduces neurological dysfunction after cerebral ischemia/reperfusion by downregulating NLRP3 inflammasome components (NLRP3/ASC/caspase-1/GSDMD/IL-1β/IL-18) [191]. Remimazolam upregulates Nrf2/HO-1 signaling via α7nAChR/vagus nerve pathways—attenuating oxidative stress injury/neuroinflammation/cognitive dysfunction after LPS challenge in rats [189]. Remimazolam reduces HMGB1 protein expression in septic rat lungs with ARDS—correlating with decreased proinflammatory cytokines (IL-1β/IL-6/TNF-α) and improved function [192].

#### 6.3.2. Translational Biomarker Evidence

In ICU patients with ARDS, it has been reported that receiving midazolam-based sedation suppresses plasma levels of IL-1β (~21%), IL-6 (~21%), TNF-α (~19%), and IL-8 (~48%) after 48h infusion compared to baseline, whereas propofol increases these cytokines except for IL-8 which is also suppressed by both agents [176,180]. Midazolam ameliorates LPS-induced BBB disruption by increasing ZO-1 expression and reducing permeability via RhoA/ROCK2 pathway inhibition, in both mouse models/in vitro endothelial cells, accompanied by reduced MDA and increased SOD activity [197]. Conversely, remimazolam besylate reduces cortisol more than dexmedetomidine at 48h post-administration. It lowers anti-inflammatory cytokines IL-4/IL10 compared to dexmedetomidine but has less effect on IL-6 [124]. However, it seems that combined sedation strategies including midazolam further lower inflammatory cytokines compared to single agents alone [180].

#### 6.3.3. Observational Clinical Evidence

High systemic inflammation, in cases of elevated IL-6 for example, reduces midazolam clearance in COVID-related ARDS patients. This raises the risk for oversedation and delirium but also reflects altered immune–metabolic crosstalk during hyperinflammatory states [183]. Prolonged deep sedation with benzodiazepines is associated with increased delirium days post-critical illness, but direct links to specific molecular pathways remain limited.

### 6.4. Ketamine

Ketamine is an anesthetic derived from phencyclidine that exerts its effects primarily through non-competitive antagonism of N-methyl-D-aspartate (NMDA) receptors. It has demonstrated significant utility in the management of acute brain injury, where the primary objectives are to limit the extent of primary injury and prevent secondary injury mechanisms. In this context, ketamine has been associated with neuroprotective effects that extend beyond its anesthetic properties, including anti-inflammatory and anti-apoptotic actions [242].

Evidence from animal models, translational biomarker studies, and limited clinical data suggests that ketamine reduces systemic and neuroinflammatory cytokines (TNF-α, IL-1β, IL-6), inhibits key signaling pathways such as NF-κB and NLRP3 inflammasome activation, decreases oxidative stress/ROS production, and may protect against cognitive dysfunction associated with ARDS or critical illness [206,211,213,215,218,219,220,225,229,230,233]. Mechanistically, ketamine’s actions span the HMGB1/RAGE/NF-κB axis, the TLR4/MAPK/ERK1/2 pathways, Akt/mTOR signaling, and modulation of macrophage polarization toward anti-inflammatory phenotypes [206,219,228,229,230]. However, direct evidence in ARDS patients remains sparse, with most findings being derived from animal models or extrapolated from related critical illness states.

#### 6.4.1. Preclinical Mechanistic Evidence

Animal/in vitro studies consistently show that ketamine reduces pro-inflammatory cytokines (TNF-α, IL-1β, IL-6) in both ALI/ARDS and neuroinflammatory models. Ketamine inhibits NF-κB activation, including p65 phosphorylation, suppresses NLRP3 inflammasome activity which reduces IL-1β levels, downregulates TLR4/MAPK/ERK1/2 signaling [206,211,213,218,219,225,228,229,230], and promotes autophagy-mediated anti-inflammatory responses [215]. It also shifts macrophage polarization toward M2 anti-inflammatory phenotypes via mTOR/Akt signaling [228].

Preclinical studies show that ketamine reduces ROS production while increasing antioxidant enzymes (SOD/CAT/GSH) via Nrf2 pathway activation [206,224]. In LPS-induced delirium and neuroinflammation models, R-ketamine attenuated both systemic inflammation and cognitive deficits [227]. However, meta-analysis indicates acute cognitive impairment with high-dose ketamine. Chronic use is associated with structural brain changes and cognitive decline [199,203], while a low-dose strategy may be neuroprotective postoperatively or after brain injury [206,224].

#### 6.4.2. Translational Biomarker Evidence

Translational studies in humans with depression or perioperative settings demonstrate reductions in circulating TNF-α, IL-6, IL-1β after ketamine administration [201,202,218,219,226]. In a clinical trial of acute lung injury patients related to mechanical ventilation, ketamine reduced serum NF-κB activity and inflammatory markers. Esketamine also decreased IL-6 levels postoperatively [233].

#### 6.4.3. Clinical Observational and Randomized Evidence

Clinical data directly from ARDS populations are limited. One pilot study found that both inhaled and infused ketamine improved ventilatory parameters but did not report detailed biomarker outcomes [210]. In COVID-19 ARDS patients requiring sedation, adjunctive ketamine did not significantly alter mortality but was used more often in sicker patients [198]. Meta-analysis of cardiac surgery RCTs showed modest reductions in perioperative IL-6/CRP with ketamine but no improvement in clinical outcomes [214].

Conflicting findings arise mainly around dose and duration. High or prolonged dosing can induce cognitive impairment and neurotoxicity, while subanesthetic doses appear protective or neutral regarding cognition, especially when used acutely or perioperatively rather than chronically [199,203].

### 6.5. Thiopental

Recent work focuses on thiopental mainly as an anesthetic with specific niche uses, neurocritical care for example, rather than as a routine ICU sedative or standard ARDS drug. Most current ICU sedation protocols favor propofol, midazolam, and dexmedetomidine [6,7,243].

Thiopental has been shown to exert immunomodulatory effects [32,33], direct [239] and indirect [234,235] anti-inflammatory properties, as well as downregulation activity of neuronal injury biomarkers [237,244,245]. However, the current evidence regarding the anti-inflammatory and immunomodulatory effects of thiopental is limited and derived primarily from small-scale experimental and clinical studies.

Overall, none of the recent papers describe thiopental as part of standard ARDS sedation protocols or as a frontline ICU sedative in mechanically ventilated ARDS. Contemporary practice trends and large observational data indicate thiopental is rarely used [12,246] compared with propofol, midazolam, and dexmedetomidine.

## 7. Discussion

In this review we covered the most common intravenous sedatives used both in ARDS and critical illness as well as in neurocritical care that may provide several other effects other than anesthesia, such as anti-inflammation and neuroprotection within the lung–brain axis in acute respiratory distress syndrome. Emerging evidence suggests the existence of bidirectional communication between the lungs and the brain, mediated through complex signaling pathways and the release of inflammatory mediators. Within this framework, we hypothesized that specific sedative agents may modulate lung–brain signaling pathways, thereby attenuating both pulmonary injury and neuroinflammation, as well as the neurocognitive impairment associated with acute respiratory distress syndrome.

The evidence supports that dexmedetomidine exerts anti-inflammatory effects via α2-adrenergic receptor-mediated inhibition of pro-inflammatory cytokines (TNF-α↓, IL-6↓, IL-8↓), suppression of NF-κB signaling (including HMGB1/RAGE/NF-kB axis), downregulation of NLRP3 inflammasome activity (IL-1β↓), modulation of macrophage polarization toward M2 phenotype via AMPK/SIRT1 pathway activation [127,144], reduction of oxidative stress (ROS↓/SOD↑) [130,131], preservation/restoration of autophagic flux [140], inhibition of ERK1/2 phosphorylation leading to increased microglial M2 polarization [139], and attenuation of astrocyte pyroptosis/neuroinflammation/cognitive impairment via multiple axes including miR340/NF-kB/c-Fos/NLRP3/caspase cascades [133,247].

Propofol exerts broad anti-inflammatory effects across the lung–brain axis by targeting key molecular pathways involved in both pulmonary inflammation, for example NLRP3 inflammasome inhibition via Nrf2 activation, and neuroinflammation, such as suppression of microglial activation via PI3K/Akt/mTOR/HIF-1α downregulation [107,157,164]. These actions translate into reduced production of pro-inflammatory cytokines (TNF-α, IL-1β), decreased oxidative stress generation by neutrophils and myeloperoxidase inhibition [174], preservation of BBB integrity through MMP-9 suppression [171], attenuation of neuronal apoptosis via ferroptosis inhibition [164], maintenance of Th17/Treg balance via miRNA regulation [173], and improved cognitive outcomes post-injury or surgery. However, the directionality is context-dependent. While most preclinical data show consistent anti-inflammatory and neuroprotective effects at clinically relevant concentrations [107,157,163,166,172,173], some translational and clinical studies report less efficacy compared to alternative sedatives like dexmedetomidine [155,161,170]. It is important to note that dexmedetomidine often outperforms propofol in neuroprotection and anti-inflammation activity [111,143,161], despite propofol providing a generally favorable but not universally superior effect on cognitive impairment [156]. This discrepancy highlights a potential clinical gap in propofol’s neuroprotective profile. Specifically, while preclinical models emphasize robust microglial modulation, clinical observations of elevated S100B levels suggest that its ability to maintain blood–brain barrier integrity may be less effective compared to dexmedetomidine in high-acuity ICU settings.

Numerous studies support a mechanistic rationale for the anti-inflammatory, neuroprotective, and immune-modulating effects of benzodiazepines/midazolam across the lung–brain axis, particularly through the suppression of pro-inflammatory cytokines (TNF-α/IL-6/IL-1β), inhibition of the NF-κB/MAPK/NLRP3 pathways via TSPO/PBR engagement on glial cells [178,186,193,194], reduction of oxidative stress [189,197], preservation of BBB integrity [197], attenuation of microglial activation [182], and improvement in cognitive outcomes following systemic inflammation [189,190]. These effects are consistently observed across preclinical animal/in vitro models as well as translational biomarker studies in humans with ARDS/sepsis. However, clinical outcome data remain mixed since some studies show improved oxygenation/lung function [180], and others highlight risks such as delirium due to altered drug metabolism during hyperinflammatory states [183]. The directionality is generally toward decreased inflammation/neurotoxicity when BZDs are used acutely or at moderate doses, but chronic or prolonged exposure may impair host defense or contribute to adverse neurocognitive sequelae [184,185]. Importantly, the strongest mechanistic coherence exists for TSPO-mediated suppression of innate immune responses, but direct demonstration within ARDS populations is limited but growing [180,192]. However, most findings regarding α2 adrenergic interactions are indirect or derived from comparative sedative studies rather than direct BZD action. Some conflicting results exist regarding anti-/pro-inflammatory gene expression shifts depending on context [184].

The variety of mechanistic evidence supports ketamine’s ability to suppress systemic inflammation, particularly by reducing TNF-α, IL-1β, and IL-6 via inhibition of the NF-κB/NLRP3/TLR4-MAPK pathways, effects observed robustly in animal models of ALI/ARDS as well as neuroinflammatory states [206,211,218,219,229]. These molecular actions translate into reduced pulmonary injury scores and improved histopathology in preclinical ARDS models. Similar anti-inflammatory effects are seen centrally, within the brain, with attenuation of microglial activation and neuroinflammation [220,227]. Translational human data confirm reductions in peripheral inflammatory markers after ketamine/esketamine administration but are less consistent regarding clinical outcomes or direct CNS effects outside psychiatric populations [218,219]. Clinical trials specifically targeting ARDS remain rare. Available observational data suggest safety but do not establish efficacy for mortality or ventilator-free days [198,210]. Notably, ketamine seems to downregulate the HMGB1/RAGE/NF-κB axis to reduce lung inflammation [213]. In addition, ketamine inhibits NLRP3 inflammasome activity, leading to lower IL-1β release, which may translate to neuroprotection [215]. Also, through the Akt/mTOR signaling pathway, ketamine promotes M2 macrophage polarization. Conversely, mTOR inhibition blocks some anti-inflammatory actions of S-ketamine but not R-ketamine, which may act via ERK [228]. Finally, ketamine reduces ROS/MDA while boosting SOD/CAT/GSH through Nrf2 pathway activation. These antioxidant effects likely contribute to organ protection during systemic inflammation, such as the ARDS-related one [206,224].

Thiopental strongly inhibits NF-κB in T-cells, suppressing IL-2, IL-6, IL-8, IFN-γ and lymphocyte activation [234], but in current medical practice it is no longer used.

Ultimately, the clinical implications of sedative properties remain uncertain. The relationship between depth of sedation and neurological outcomes, including delirium, is complex and not fully elucidated. Therefore, not all sedation is beneficial since deep or prolonged sedation can worsen cognitive outcomes if not carefully managed [44,113]. While some data suggest that deeper levels of sedation are associated with worse outcomes, including increased mortality and prolonged recovery in ARDS and general ICU populations [102,248], lighter sedation strategies may also be associated with adverse effects such as agitation and patient–ventilator asynchrony. These findings highlight the importance of agent selection and titration based on patient-specific factors, as current practice emphasizes individualized, goal-directed sedation strategies, including regular reassessment and minimization of unnecessary exposure [249]. Daily sedation interruption and titration to the lowest effective dose are commonly recommended approaches aimed at reducing the risk of delirium, prolonged mechanical ventilation, and other adverse outcomes [96,104].

## 8. Research Agenda

Future research should focus on large-scale clinical trials comparing different sedation strategies’ impact on both short- and long-term neurological outcomes in patients with critical illness involving both lungs and the brain. The transition from recognizing the lung–brain axis to actively modulating it requires a fundamental shift in the research agenda, moving beyond isolated mechanistic observations toward an integrated clinical framework. A priority remains the execution of large-scale, randomized controlled trials that can definitively determine whether the molecular anti-inflammatory actions of agents like dexmedetomidine and propofol—so consistently observed in preclinical models—actually translate into improved survival, more ventilator-free days, and superior neurological recovery in human ARDS cohorts. Such trials must move beyond generic outcomes, incorporating biomarker-guided stratification (S100B or NSE) to identify subphenotypes of patients who might derive the greatest benefit from early lung–brain protective sedation.

Central to this agenda is the need to clarify the dose- and timing-dependent relationships that govern sedative-mediated immunomodulation. For instance, future research must reconcile the pharmacological paradox of benzodiazepines, identifying the precise threshold where their theoretical anti-inflammatory potential (mediated via TSPO or NLRP3 inhibition) is clinically overwhelmed by their well-documented pro-deliriogenic effects. Similarly, for ketamine, the research focus should shift toward its net systemic effect, specifically evaluating how its potent suppression of the HMGB1/RAGE/NF-κB axis interacts with its sympathomimetic-mediated cerebral metabolic stress.

Looking ahead, we must also bridge the gap between acute ICU interventions and long-term quality of life. Elucidating the role of emerging pathways, such as the STAT6/IRF4-mediated macrophage reprogramming or the Akt-driven preservation of the blood–brain barrier, will be essential in developing therapies that do not merely ensure survival but actively prevent the neurocognitive sequelae of post-intensive care syndrome (PICS). By validating these animal-derived signaling mechanisms within human populations through robust translational biomarkers, the field can finally transition from a strategy of “biologically neutral” sedation to one of active, organ-specific protection.

## 9. Summary

Sedation strategies that improve pulmonary physiology, especially those including dexmedetomidine, can confer significant downstream neuroprotective benefits via modulation of systemic inflammation along the lung–brain axis. However, further clinical research is needed to optimize protocols for maximal benefit with minimal risk. Dexmedetomidine shows strong promise as an immunomodulator across the lung–brain axis, but further targeted clinical research is needed to establish its full therapeutic potential in ARDS. Propofol exerts broad anti-inflammatory/neuroprotective effects across the lung–brain axis primarily through PI3K/Akt/NF-kB/NLRP3 suppression, but further research is needed for definitive clinical translation and mechanistic clarity regarding less-studied pathways such as STAT6/IRF4. Benzodiazepines exhibit clear anti-inflammatory actions through multiple convergent molecular mechanisms relevant to both pulmonary/systemic inflammation and neuroprotection, but further translational research is needed before these findings can be fully leveraged clinically for ARDS management. Ketamine shows strong promise as an immunomodulator across the lung–brain axis, especially at the molecular level, but further translational research is required before it can be considered a clinically actionable therapy for ARDS-related inflammation or neurocognitive sequelae. Finally, thiopental has been shown to exhibit several anti-inflammatory and immunomodulatory effects that could mitigate neuroinflammation and cognitive impairment, but it is seldom employed in modern ICU sedation practice; therefore, its properties remain purely of theoretical importance.

## 10. Conclusions

Patients with ARDS represent a distinct subset of critically ill patients in whom extrapulmonary manifestations and systemic immune dysregulation significantly influence disease progression and outcomes. The interplay between pulmonary injury and systemic inflammation, including neuroinflammatory involvement through the lung–brain axis, contributes to the development of ARDS-associated encephalopathy and cognitive dysfunction. In this context, sedative agents should be regarded not only as tools for achieving patient comfort and ventilator synchrony but also as pharmacologic interventions with potential immunomodulatory and neurobiological effects. Current evidence indicates that certain sedatives—especially dexmedetomidine—can modulate pulmonary physiology by reducing inflammation/compliance issues and thereby limit cytokine spillover across the lung–brain axis to achieve downstream neuroprotection. Careful agent selection and titration is essential to maximize benefits while minimizing risks for long-term cognitive impairment.

Taken together, sedatives may represent promising modulators of inflammatory and neuroinflammatory processes relevant to the lung–brain axis in ARDS.

## Figures and Tables

**Table 1:** Comparative Synthesis of Sedative Effects on the Lung–Brain Axis.

Sedative	Model & Evidence Level	Molecular Mechanisms	Rank	Effects on the Lung–Brain Axis	Clinical Outcomes	Ref.
Dexmedetomidine (DEX)	Mostly preclinical/Meta-analyses. Robust experimental data & translational biomarkers.	α2-adrenoceptor activation; HMGB1/RAGE/NF-kB & NLRP3 inhibition; AMPK/SIRT1 activation.	Direct ARDS evidence (precl.)/ICU extrapolation (clinical)	Attenuates lung edema & microglial neuroinflammation; promotes M2 polarization; preserves BBB integrity.	Reduced S100B/NSE; lower delirium/POCD incidence; mortality benefits inconsistent.	[100,115,116,117,118,119,120,121,122,123,124,125,126,127,128,129,130,131,132,133,134,135,136,137,138,139,140,141,142,143,144,145,146,147]
Propofol	Mostly preclinical/observational. Strong experimental support; mixed translational signals.	PI3K/Akt/mTOR/HIF- α downregulation; Nrf2/HO-1 activation; MMP-9 inhibition; miRNA regulation.	Direct ARDS evidence (precl.)/general ICU extrapolation (clinical)	Inhibits microglial ferroptosis & metabolic reprogramming; maintains Th17/Treg balance; scavenges ROS.	Reduced cerebral metabolic rate; mixed S100B signals; generally favorable but not superior cognitive effects.	[99,107,111,148,149,150,151,152,153,154,155,156,157,158,159,160,161,162,163,164,165,166,167,168,169,170,171,172,173,174,175,176]
Benzodiazepines (BZDs)	Preclinical/translational biomarkers. Strong mechanistic links but clinically overshadowed by adverse effects.	TSPO/PBR engagement; inhibition of NF-kB/p38 MAPK; NLRP3 suppression; RhoA/ROCK2 inhibition.	General ICU/ARDS extrapolation	Suppression of TNF-α /IL-6 burst; reduction of HMGB1 in lungs; preservation of ZO-1 in BBB; attenuation of astrocyte/microglia pyroptosis.	Paradoxical: shows systemic cytokine reduction in ARDS; however, associated with increased delirium and prolonged ventilation.	[177,178,179,180,181,182,183,184,185,186,187,188,189,190,191,192,193,194,195,196,197]
Ketamine	Preclinical/pilot clinical. Extensive animal data in ALI and neuroinflammation; limited but growing human biomarker data.	NMDA receptor antagonism; suppression of TLR4/MAPK/ERK1/2; Akt/mTOR-mediated M2 polarization; Nrf2-driven antioxidant response.	Direct ARDS evidence (preclinical)/pilot clinical signals	Attenuates HMGB1/RAGE-mediated lung injury; reduces systemic NF- kB activity; limits excitotoxicity and microglial activation; promotes autophagy.	Dose-dependent: subanesthetic doses show reduced IL-6/CRP and improved ventilatory parameters; high/chronic doses risk neurotoxicity and structural brain changes.	[103,110,198,199,200,201,202,203,204,205,206,207,208,209,210,211,212,213,214,215,216,217,218,219,220,221,222,223,224,225,226,227,228,229,230,231,232,233]
Thiopental	Primarily in vitro. Strong systemic immune suppression signals in vitro; clinical use is largely restricted.	Selective NF-κB inhibition; potent scavenging of ROS (superoxide, NO, hydroxyl radicals).	In vitro mechanistic/rescue therapy	Potent systemic immunosuppression (reduced IL-2, IL-6, IFN-γ); lowers ICP and cerebral metabolism; theoretical antioxidant organ protection.	Rescue use only: effective for refractory intracranial hypertension and ventilator asynchrony; high risk of hypotension, pneumonia, and sepsis.	[32,234,235,236,237,238,239]

Note. α-adrenoceptor, alpha-2 adrenergic receptor; AMPK, AMP-activated protein kinase; BBB, blood–brain barrier; CRP, C-reactive protein; ERK1/2, extracellular signal-regulated kinase 1/2; HMGB1, high mobility group box 1; HO-1, heme oxygenase-1; IL, interleukin; MAPK, mitogen-activated protein kinase; MMP-9, matrix metalloproteinase-9; mTOR, mammalian target of rapamycin; NF-kB, nuclear factor kappa B; NLRP3, NOD-like receptor protein 3; NMDA, N-methyl-D-aspartate; NSE, neuron-specific enolase; RAGE, receptor for advanced glycation end-products; ROS, reactive oxygen species; SIRT1, Sirtuin 1; TLR4, toll-like receptor 4; TNF-α, tumor necrosis factor-alpha; ZO-1, zonula occludens-1.

## Data Availability

No new data were created or analyzed in this study. Data sharing is not applicable to this article.

## Abbreviations

The following abbreviations are used in this manuscript:

Akt	Protein Kinase B
AP-1	Activator Protein 1
ARDS	Acute Respiratory Distress Syndrome
BBB	Blood–Brain Barrier
CRP	C-Reactive Protein
ERK	Extracellular Signal-Regulated Kinase
GABA	Gamma-Aminobutyric Acid
HIF-1α	Hypoxia-Inducible Factor 1-alpha
HMGB1	High Mobility Group Box 1
ICU	Intensive Care Unit
IL	Interleukin
IRF	Interferon Regulatory Factor
MAPK	Mitogen-Activated Protein Kinase
MIP	Macrophage Inflammatory Protein
MMP	Matrix Metalloproteinase
mTOR	Mammalian Target of Rapamycin
NETs	Neutrophil Extracellular Traps
NF-κB	Nuclear Factor kappa B
NFL	Neurofilament Light Chain
NLRP3	NOD-Like Receptor Protein 3 Inflammasome
NMDA	N-Methyl-D-Aspartate
NO	Nitric Oxide
NSE	Neuron-Specific Enolase
PI3K	Phosphoinositide 3-Kinase
RAGE	Receptor for Advanced Glycation End-Products
ROS	Reactive Oxygen Species
S100B	S100 Calcium-Binding Protein B
STAT	Signal Transducer and Activator of Transcription
TLR4	Toll-Like Receptor 4
TNF-α	Tumor Necrosis Factor alpha
VABI	Ventilator-Associated Brain Injury
VILI	Ventilator-Induced Lung Injury
